# Assessing people with dementia participating in cognitive stimulation activities—A qualitative pilot video analysis exploring the importance of facilitating the participation

**DOI:** 10.1111/hex.13300

**Published:** 2021-06-11

**Authors:** Diana Schack Thoft, Anna Camilla Ottesen, Anne Melchior Jensen, Alison Ward

**Affiliations:** ^1^ University College Northern Denmark Aalborg Oest Denmark; ^2^ University of Northampton Northampton UK

**Keywords:** dementia, measures, facilitation

## Abstract

**Background:**

This pilot video analysis was part of a feasibility control study, which aimed to gain information about the size and variability of the changes in outcome measures to plan a substantive effect study. It compared a cognitive stimulation programme named Lifelong Learning with other existing dementia services.

**Objective:**

The pilot video analysis explored how facilitation is performed, when assessing people with dementia with standardized measures, to ensure their participation in research.

**Design:**

A test battery of five measures (Mini‐Mental State Examination (MMSE), Quality of Life in Alzheimer's Disease Scale (QoL‐AD), General Self‐Efficacy Scale, Rosenberg Self‐Esteem Scale and Hawthorn Friendship Scale) was used. Each assessment was video‐recorded. The findings from a microanalysis of 10 videos are presented in this article.

**Setting:**

The study involved 55 active participants with mild‐to‐moderate dementia in six municipalities in Northern Denmark.

**Results:**

The identified themes related to supportive facilitation: *Positive facilitator strategies; Creating a safe and comfortable environment*; and to dilemmas in facilitation: *Balancing multiple dilemmas* and *Balancing the MMSE test*.

**Discussion:**

Results are discussed in relation to using standardized measures.

**Conclusion:**

The quality of facilitation when using standardized measures is of great importance as it may influence the participant, the assessment and the answers given. The facilitation role needs to be thoroughly planned and executed with ethical consideration to improve the participation of vulnerable groups in research and ensure a person‐centred approach.

**Patient or public contribution:**

The identified measures were chosen based upon previous qualitative results and user‐involvement workshops with people with dementia.

## 1 INTRODUCTION

Dementia research has developed over time with various areas channelling the focus of research to explore causes of dementia, treatment, understanding and relational aspects of dementia care.^1^ ‘Treatment‐oriented’ research explores different types of treatment, including how they minimize any decline or support maintenance of a person's condition.^1^, ^2^ Following a review of non‐pharmacological treatment for people with Alzheimer's disease, Cammisuli et al^3^ identified four categories of non‐pharmacological treatment: holistic techniques; brief psychotherapies; cognitive methods; and alternative strategies (p.58). The intervention described in this paper, that of Lifelong Learning, fall under the holistic definition,^3^ which includes reminiscence, reality orientation and cognitive stimulation.

Harding et al^4^ suggest evaluations for non‐pharmacological interventions have limitations in relation to the way they are defined, which make it challenging to evaluate and compare findings. Webster et al's^5^ review of outcome measures found 81 measures used by 125 studies, demonstrating great heterogeneity of measures used. With the use of different outcome measures, models and duration of the interventions, it becomes difficult to compare across studies and to compare with drug trials.^6^, ^7^

Furthermore, Harding et al^4^ comment on the lack of agreement between professionals and people with dementia as to what is important to measure and how. It may be relevant to measure psychosocial interventions in different ways as these may not be properly measured by commonly used psychometric measures.^8^, ^9^, ^10^, ^11^ It can be challenging to identify what measures to use and whether qualitative methods may provide a valuable perspective. The inclusion of people with dementia is a valid addition, in terms of understanding of the impact and the relevance of interventions from a personal perspective.^4^, ^12^, ^13^ Harding et al^4^ discuss how their voice often is absent in decisions about what research should focus on when evaluating and, hence, which outcome measures to use. This tends to remain within research, but value can be gained from discussing and aligning the research outcomes with issues important to those with dementia.

Another aspect that is rarely discussed is how participants with dementia experience an assessment. Dementia is a progressive cognitive condition. When considering the design of research with people with dementia, the impact on that person's memory, decision making, understanding, concentration, mood and problem‐solving may be pertinent to ensure the assessment does not become a burden,^5^, ^14^ causing fatigue or distress, as they may have difficulties in paying attention for a longer period.^15^ The design may also be a barrier to participation if not all measures are completed.^6^, ^16^, ^17^ Furthermore, when using measures with people with dementia, ethical factors, such as ways of supporting vulnerability and dignity, are relevant to consider.^18^ This influences the identification of reliable and appropriate measures.^5^

A recent review of cognitive stimulation interventions identified research uses 5‐6 measures on average, but this can be as many as 10‐15 in a single study.^19^ No comment is made in these studies as to what the appropriate number of measures is to use, and little is stated about the relevance of the measures chosen or how the person with dementia experiences them.

Furthermore, little information is provided on how assessments are conducted in accordance with the instructions,^20^ how the tests are administrated and whether the total time of the battery is taken in consideration to minimize the burden for participants.^20^, ^21^ It is therefore difficult to judge the quality of the assessments, which may potentially influence the quality of the results. This paper trials a new approach, using video recordings, to explore the facilitation of measures in the assessment process, providing new insight into this little‐studied aspect of conducting research together with people with dementia to ensure their participation in research.

## 2 METHODS

This paper presents findings from a pilot video analysis, which was part of a feasibility study, to gain information about the assessment of a Lifelong Learning Service for people with dementia. The study compared an intervention group receiving Lifelong Learning with a control group, participating in treatment as usual (services at day‐care centres, etc). The study was conducted in six Danish municipalities. The Lifelong Learning concept is an on‐going cognitive stimulation programme, aiming to support cognition, decision making, activities of daily living and social engagement.^22^, ^23^, ^24^, ^25^, ^26^ The study followed the Standards for Reporting Qualitative Research (SRQR checklist for qualitative studies).

### 2.1 Recruitment

Recruitment was supported through the staff at each of the services, who were guided on the study´s inclusion criteria that participants should: have a dementia diagnosis; participate in a service; and be able to consent. In total, 88 participants were recruited for the feasibility study. The dropout/exclusion rate was 37.5% due to progression of dementia, hospital admission, relatives’ illness, non‐dementia diagnosis and death. Participants were excluded if they attended less than 10 sessions. This resulted in 55 participants (n = 30 intervention group and n = 25 control group) with a median age of 76 years and MMSE (mean = 18.44, SD = 5.16) in the control group and 72.5 years and MMSE (mean = 21.83, SD = 3.43) in the intervention group. Most participants had a diagnosis of Alzheimer´s disease.

### 2.2 Measures

The measures were identified by a user‐involvement workshop with people attending the lifelong service and interviews with service staff. This guided the choice of the measures used, which were the: Mini‐Mental State Examination Test (MMSE‐2)^27^, ^28^; Quality of Life in Alzheimer's Disease Scale (QOL‐AD)^29^, ^30^; General Self‐Efficacy Scale^31^; Rosenberg Self‐Esteem Scale^32^; and Hawthorn Friendship Scale.^33^ The assessments were facilitated by first, second and third author, all with backgrounds in nursing.

### 2.3 Video analysis

Each participant was assessed pre‐ and post‐intervention, over 5‐6 months. Each assessment was video‐recorded. An adapted^25^ version of Ridder's^34^ video analysis was used for the data analysis. The aim of this pilot analysis was to answer the research question: *How is facilitation performed, when assessing people with dementia with standardized measures, to ensure their participation in research?*

During the analysis phase, it was important to consider what the participants responded verbally and non‐verbally. The rationale for using video recordings was to ensure these nuances of social interaction were captured. The value of video is the ability to watch and re‐watch interactions in a way that observation alone does not enable.^35^

After reviewing all videos (n = 55 pre‐assessment and n = 55 post‐assessment), a stratified sample (videos divided into subgroups based upon characteristics) of ten videos (n = 10) was used to include the following: pre‐assessment videos; five control and five intervention participants; example from each site; level of dementia (high and low MMSE score); and diversity of gender. The ten videos were chosen based upon a team discussion of their characteristics (gender of the participant, control/intervention group, level of dementia and response to measures), including a review of notes and summaries of the videos, with a focus on the research question. The inclusion criteria were based on providing representation across the data and not only using examples from high‐functioning individuals or from one intervention group. Only pre‐assessment videos were chosen, as these would show the participants’ first encounter with the measures (see Table 1) to avoid recall or familiarity with the measures.

**TABLE 1:** Details related to the video chosen

Video No.	Gender	Type of dementia	Age	Region: setting	Intervention/control	Video length
7	Male	AD	74	Region 1: Service	Control	57 min 37 s
31	Male	AD	77	Region 2: Service	Control	25 min 54 s
34	Male	OTHER	73	Region 3: Service	Intervention	58 min 30 s
49	Female	OTHER	65	Region 4: Service	Intervention	31 min 31 s
55	Male	Not specified	89	Region 4: Home	Control	37 min 30 s
60	Female	AD	62	Region 2: Service	Intervention	37 min 5 s
71	Male	OTHER	54	Region 5: Service	Control	25 min 49 s
75	Male	OTHER	74	Region 6: Service	Intervention	22 min 33 s
82	Female	OTHER	68	Region 7: Home	Control	33 min 52 s
86	Female	AD	76	Region 4: Service	Intervention	25 min 45 s

**FIGURE 1:**
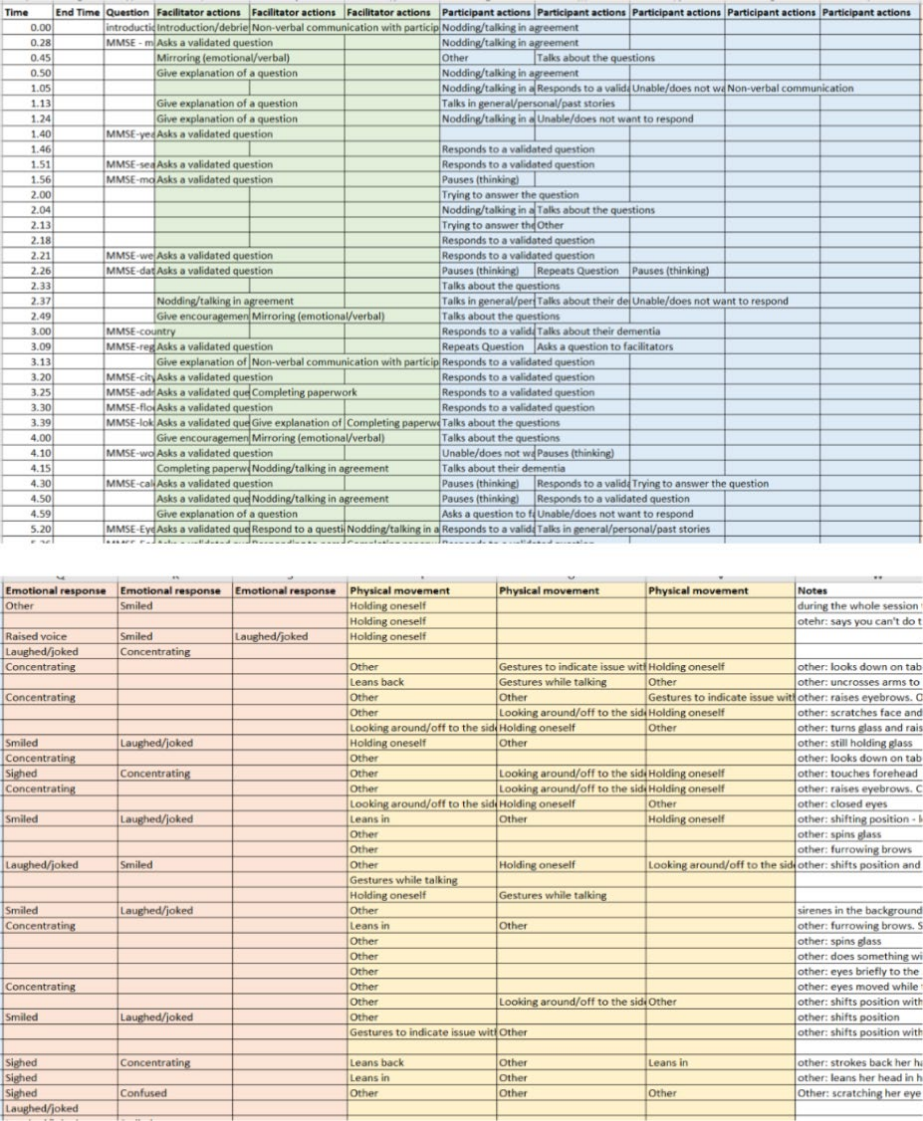
Example of video graph

All videos were watched multiple times to identify codes for a video graph. An analysis framework was developed and tested using one video. This framework was adapted with additional coding options included for the remaining videos to identify moments with facilitation.^34^, ^36^, ^37^ A graph was created for all videos to log each interaction and questions asked, and identify moments for a deeper analysis (see Figure 1).

The video graph enabled the selection of clips for a microanalysis. Notes had been made on the graph of moments, which showed typical/atypical situations of facilitation.^37^ Therefore, 13 clips were chosen. The length of the clips reflects that interactions were often short, with participants responding quickly (see Table 2).

**TABLE 2:** Video clips included in microanalysis

Video No.	Duration of clip	Description of clip
7	25 s	Participant experiences uncertainty in responding (MMSE)
31	17 s	Participant's disappointment in responding to the measure
34	45 s 24 s	Example of strategies used by the participant (MMSE) Participant's emotional response (QoL)
49	3 min and 5 s	Example of facilitation (Self‐Efficacy)
55	43 s	Participant explains about his hearing disability (MMSE)
71	1 min 30 s	Participant shows challenges in answering (Self‐Efficacy)
75	1 min and 31 s	Participant has difficulties in answering (MMSE)
	25 s	Participant being in a good mood (Self‐Efficacy)
82	1 min	Participant discusses the term ‘excellent’ (QOL)
86	3 min	Participant is not aware of the dementia (QoL)
	1 min and 49 s	Example of humour used in the assessment process (MMSE)

Ridder's^34^ microanalysis process was as follows: watching each clip to get an impression; identifying ‘meaningful events’ and writing what is seen and heard; writing a ‘subjective assessment’; writing a reflection; and writing an evaluation. A section was added to allow quotes to be included.^25^, ^34^ Table 3 shows an example of this microanalysis. The final analysis stage was to draw the themes from across each microanalysis and the video graph.

**TABLE 3:** Example of the microanalysis

Video No. 86; (1.38‐3.27): Meaningful event: MMSE—repeat 3 words	Assessment of event *‘I feel/think…’ or ‘The participant seems to…’*	Reflection of event *How can you see this response, emotions, engagement, interactions?*	Supporting text *Transcription of clip*
Researcher (R) asks Participant (P) to repeat three words. R is looking at P as she speaks, and P looks at R. R and P then both look at the paper. P says yes and responds correctly straight away.	It seems a relaxed start to the MMSE. P looks as though she is concentrating and repeats the words quickly, this could be a way of not forgetting them!	There is a small space between R and P, which may make the paper more obvious in the situation and for P to see what R is writing.	

## 3 ETHICS

The Ethics Committee of Northern Denmark was informed about the study. It was judged that no further application was needed in relation to LBK nr 1083 of 15/09/2017 definition of a Health Science Research Project and the Committee law § 14, stk. 1, jf. § 2, nr 1‐3. The Helsinki Declaration was the ground upon the study was conducted.^38^

All information material and consents were developed based upon the author's experiences of producing accessible documents for people with dementia.^23^, ^25^ This allowed the participants to be informed of the study prior to agreement to participate to sign the consent themselves in collaboration with their relatives or service staff. Furthermore, an on‐going consent inspired by Dewing^39^ was used at the post‐measurement to ensure continued participation. Due to the requirements of confidentiality and anonymity, the video recordings are not allowed to be shown. All names used within the article are pseudonyms.

## 4 FINDINGS

The identified themes of *Positive facilitator strategies* and *Creating a safe and comfortable environment* related to supportive facilitation determined by enabling the participants to concentrate and give their voice, whereas the themes of *Balancing multiple dilemmas* and *Balancing the MMSE test* related to dilemmas in facilitation identified by difficulties occurring in the assessment.

### 4.1 Positive facilitator strategies

This theme revealed that the manner of interactions between the facilitator and the participant could be influential, in providing support through verbal/non‐verbal communication or in the way instructions were given. The facilitators introduced the measures, as per the instructions. Each measure had different instructions, which resulted in the support shifting as the assessment progressed. The order of the measures was also considered as some were perceived to be more challenging than others. The most difficult one was planned at the beginning (MMSE), so that the assessment became easier as it progressed, ending with the shortest measure (Hawthorne Friendship Scale).

The facilitators tried to minimize any stress in the situation by using supportive communication and giving time for participants to understand and respond. Non‐verbal support included the following: eye contact, active listening, nodding, giving pauses and pointing on the test paper to draw attention to the question. Verbal communication included the following: validation of the participants’ emotions and responses, ensuring the participants’ answers were correctly understood, and repeating and explaining questions.

The positive facilitation seemed to include for example to support participants to choose the answer they felt most appropriate. Sometimes, the facilitator needed to translate the responses to ensure their answer correlated with the response categories. In order not to misinterpret a response, the facilitators adopted a strategy to first identify whether the participant agreed or disagreed with a question and then to ask by how much. In doing this, the facilitator helped the participants to focus on two options rather than four, as exemplified when Lone was asked the question: ‘*I feel I do not have much to be proud of’* in the Rosenberg Self‐esteem Scale:

If you are **not** proud of anything, then you could say you are over here where you are agreeing (Facilitator points to the agreement points) If you feel you **are** proud of something, then you disagree with the sentence, you could say (Facilitator points to the disagree points)

The facilitator helped Lone to break down the required answer into a step‐by‐step process that was manageable and used the response paper as a visual aid. The highlighted words indicate where the facilitator placed emphasis on words to support the distinction between agreeing and disagreeing.

Another facilitating strategy was to explain that there were no right or wrong answers, with the aim to minimize any pressure in the situation. However, for the MMSE, participants sensed there was a right or wrong answer, and facilitators found that reassurances enabled participants to recognize that it was okay if they could not remember, thereby reducing perceived stress during this assessment. Facilitators responded when participants asked for help or reassurance to understand or respond to a question. The facilitators reflected that it was ethical to do this, as the participants showed trust in them by asking for help. However, not all the measures enabled this support. The Danish MMSE guidance based upon the Folstein et al^40^ user guide states that it is not allowed to: repeat questions; correct mistakes; or help the patient with the tasks.^41^

### 4.2 Creating a safe and comfortable environment

Overall, the assessment seemed quite relaxed with a positive atmosphere. The facilitators leaned forward towards the participants, creating an intimate space, and gave positive reinforcement. This was done by nodding, validation and agreeing with the responses even where it was not a direct answer to the questions asked. Often, the facilitator and the participant enjoyed a cup of coffee, which was used to create small breaks, reducing the level of formality.

Participants were observed to ask questions about the measures, and they also shared stories about home and family life in response to the assessment questions. This created a more conversational style of interaction, which the facilitators were seen to follow. This approach seemed to make a relaxed space that supported the way the participants responded. However, this was not always possible, particularly during the MMSE where personal stories and breaks were not encouraged because the guidance stipulated there should have no disturbances and answers should be provided within ten seconds.^41^

In some situations, the facilitators needed to manage frustrations and anger expressed by the participants, acknowledging the participants’ emotions when talking negatively about their lacking abilities due to their dementia. During the QoL‐AD, Hans shared his frustration about how it had become difficult to remember:

It is incredible how much… it is gone… I cannot remember what the hell things are called… (points towards the coffee pot. Speaks faster with an agitated voice) I try everything (articulates loudly) … but I am an idiot. Sometimes, I laugh of myself (looks down and towards the facilitator). Oh, the hell how ridiculous you are… (pulls a funny face) (Video 34)

The participants seemed comfortable in expressing their emotions, and facilitators managed both positive and negative emotions, by showing empathy, giving space to process emotions and listening to their stories.

This was demonstrated in the way the facilitators gave positive comments about the process, for example commenting on the number of measures completed and how the participant was doing well. Although the assessment sometimes became tense, it did not seem to influence the situation negatively. Here, the facilitator's role was important in validating the participants to keep the atmosphere relaxed, which the facilitators reflected was important.

Humour and laughter were used by both parties. Sometimes, it was used by the facilitators as a way to reduce the power balance, by making fun of themselves if they fumbled over a word or muddled up the paperwork. On other occasions, it showed moments of joy and supported personal interactions. Joking was also used as some questions could be perceived as childish (eg pointing at the eye and ear in the MMSE). Humour was also used to mirror and validate the participants’ reactions. However, laughter could also contrast with the body language and facial expression:

Oh, that yeah, yeah this is (leans back, looks to the side and back at the facilitator, laughing, smiling, pause) that is a bit difficult … I will say that we just look at eh what is it called? … yeah, the calendar and see what it says. (looks down, serious tone, tapping one finger at the table, fidgeting with his glasses) (Video 7)

In this situation, Bo used laughter to compensate his difficulties responding to the MMSE question about the year. His non‐verbal signals changed as he talked. He laughed and smiled when he had difficulties with answering and became more serious and concentrated when explaining how to answer the question.

### 4.3 Balancing multiple dilemmas

Multiple dilemmas were identified in the facilitation. Some facilitators were seen to provide their own interpretation to some of the questions when supporting the participants to respond, thereby risking leading to a specific answer. This arose out of a dilemma between supporting and leading, which may have influenced participant's responses. On occasions, a facilitator could interrupt a participant's reflection, with their own interpretation. The following example follows the question *‘If I’m in trouble I usually find a way out’* from the General Self‐Efficacy Scale:

It is true that I can. Therefore… (Bente, pausing)

Yes, hardly true? (Facilitator gestures with her hand)

Yeah, I will say moderately true, because when I can’t, then I find something else, someone that can… (Bente)

Yes, yes… So moderately true? (Facilitator)

Yes. I can find a way out if that’s what is meant. (Bente) (Video 49)

Here Bente showed that the interpretation the facilitator offered was not correct from her perspective. She was comfortable about her own judgement and gave an answer that was correct for her. However, with a different participant, this might have led to a changed answer, which illustrates the dilemma of when to give time to answer and when to support the participant. Interruptions could disturb and influence a participant's response, but conversely, not stepping in when a participant showed signs of distress could leave them frustrated. In some situations, the facilitators were observed to intervene too quickly, thinking the participant was not able to answer. Knowing when to be supportive and to maintain a participant's dignity while keeping to the guidance of the measures was challenging.

Another dilemma was also how to handle the participants’ awareness of their own difficulties. One facilitator tried to play down this situation by taking responsibility for a participant's challenges:

I was sitting thinking, I should remember them, but I can’t! (Poul)

No, that is because I have tried to confuse you (Facilitator smiles, humours tone) (Video 7)

Here, the facilitator tried to take the pressure away, as Poul was not able to recall the three words mentioned in the MMSE. This highlights the balance of administering the measures as the facilitator tried to ease the situation by deflecting the challenge of recalling the words. However, the facilitator was aware of the importance of keeping focus in the situation, and as a result, she did not encourage a dialogue about Poul's experience of dementia, but carefully moved to the next question. This highlights the dilemma of balancing the facilitation ethically and supportively, acknowledging the participants’ stories and maintaining focus of each measure's guidance.

### 4.4 Balancing the MMSE test

The power dynamic between the facilitators and the participants also seemed to change with the different measures. The MMSE was more formal, influencing the facilitators to be official in their approach. The facilitators often looked at the paperwork, wrote down responses and read the next question, almost using the paperwork to ‘hide behind’. The scoring paperwork was not shared with the participants, but often they showed interest in this, with their attention drawn to what was being written. This contrasted with the other measures, where the answer sheet was shared.

During the MMSE, there were fewer observed instances of eye contact, and shared stories about home, family life and life with dementia. Fewer instances of participant support were provided, leading to a sense of unease, by the facilitators, when administering the MMSE. It made the situation seemingly uncomfortable, as some participants showed signs of distress, by joking about their lack of skills, fidgeting or seeking intense eye contact. This was made more complex as the facilitators also found it difficult to keep track of time by only giving ten seconds per answer.^41^ As an example, Bo was observed to be uncomfortable and insecure during the MMSE, looking to the side, biting his lip and laughing to hide the challenges he faced. The situation became tense as the facilitator was not able to support him. The facilitator explained this to Bo by clarifying ‘*I’m afraid I can't* *help you’*. This could make the participants feel insecure, unacknowledged and uncomfortable, as they were confronted with their cognitive challenges.

Furthermore, the guidance on the MMSE does not allow for people to give ‘half’ answers. Facilitators had to manage this, knowing they could not give a score. Participants showed awareness of the questions posed, but did not always give an exact answer; for example, Lone answered the month instead of the season. While this was marked as an incorrect answer, Lone showed her awareness of the time of the year. Furthermore, the facilitator had to be aware not to show the participant whether they had given a correct or incorrect answer, and give limited feedback in line with the instructions.^41^ However, this could put a strain on the facilitator, who wanted to support those who were showing signs they were struggling.

The facilitators experienced needing to carefully assess how best to react during the measurement. At times, the facilitators repeated a question, when there had been an extended pause, or the participants asked the question to be repeated. They knew this meant a lost point but felt that refusing to repeat the question could add discomfort for some, especially those already exhibiting signs of anxiety. This could be criticized for disregarding the MMSE test.

## 5 DISCUSSION

This study provides insight into the process of administering validated measures and exploring the role of facilitation. This study found that participants could be supported to engage with the measures and that the role of the facilitator was important, for example, in simplifying the response scale. The structure of the response scale has been reviewed as a way to support people with dementia to engage in research using validated measures. Morbey et al's Delphi study^42^ identified that a 3‐point scale was more accessible. They found it was preferable to use a scale that asked participants the importance of a situation, rather than relying on extreme responses. Morbey et al^42^ also found the design of the questionnaire, for example the use of colour, layout, font size and use of symbols, influenced on how a person with dementia responded. These factors may be relevant to incorporate in standardized measures, many of which are not yet taking this into consideration. As this study found, participants were drawn to the paperwork and making this more dementia‐friendly could be a way to support their engagement and participation.

The MMSE used in this study was found to be challenging for both the participants, in providing the right answers within a limited time, and the facilitators, allowing limited support and feedback for the participants. In some situations, the participants showed signs of agitation. This is also identified by Hellström et al,^43^ who discuss how cognitive assessments can be humiliating and distressing, as people with dementia can feel a loss of dignity by taking the test. Such assessments tend to focus on deficits rather than strengths, which may have a negative influence on a person's self‐esteem.^43^, ^44^, ^45^ Participants in our study commented on their loss of memory throughout this test, indicating an awareness of own deficits. Webster et al^5^ recommend the inclusion of contextual, qualitative information, to provide background information on individuals, while Hellström et al^43^ identify that cognitive assessments do not leave space to talk about experiences or abilities. Taken on its own, a memory test can be demoralizing as it may be distressing to see the score and performance worsen. Participants in this study were observed to show greater signs of distress during the MMSE, adding support to the challenge of taking part in and conducting cognitive assessments with people with dementia.

Furthermore, during other assessments in this study, participants started to share stories and time was made between questions for this dialogue, highlighting the potential for greater integration of qualitative information within such assessments, as Webster et al^5^ suggest. However, it was not always possible to follow up on these comments within the guidance of the test, especially the MMSE. Criticisms of the MMSE have also been made regarding the scoring,^46^, ^47^, ^48^ which, as this study found, does not allow for related abilities to be scored. Nevertheless, the MMSE has been found to be reliable in showing decline^5^ and has become one of the most used cognitive assessments in dementia research.^48^ These contradicting findings make it challenging to identify the right measure for assessing cognition. In particular, as this research highlights, the act of administering can also be difficult.

During the study, it became obvious that in the moment of doing the assessment, time could be difficult to judge, and it could feel like a long time watching a participant trying to answer. Giving time for the person to respond is important. It is about ‘keeping to dementia time’ and working to their pace.^42^ This study found that by offering support too early, it could potentially lead to fewer points scored. On the other hand, the facilitators wanted to react and relate to the participants. Often, people with dementia are characterized as being vulnerable,^23^, ^25^, ^49^ which may affect a person's sense of well‐being and dignity,^49^, ^50^ and while the facilitators in this study did not want to play into this stereotype, they nonetheless wanted to ensure they responded in a person‐centred way that supported the participant's well‐being and dignity. Several examples of this were identified in this study, through the use of supportive communication and validating responses. However, the role of the facilitator is not often discussed in the literature. While some training is offered to complete validated measures, and guidance was provided for all the measures used in this study, these do not usually cover how to respond when a person becomes agitated during the assessment.

Morbey et al^42^ also found that the use of examples to illustrate accessible statements may not always be helpful as it can be restrictive. Accessible statements are often better left open to reflect on meaning. This was also identified in the study as the facilitators could interpret a question in a certain way and suggest a possible answer, not corresponding to how the participant had understood it. This shows the importance of not leading to an answer while giving examples that might not suit the participants. Webster et al^5^ also discuss the importance of questions being clearly worded and delivered as this may affect participants’ answers. Good communication includes reminders about discussions and rationale for the way a measure is completed. This is reminiscent of the way the facilitators in this study gave feedback on what measure was next and tried to include the participants in the process.

The vulnerability and dignity of people with dementia is essential to address in research,^43^, ^51^ and that human and legal rights are considered.^45^ According to Nordenfelt,^45^, ^52^ dignity is closely related to social relationships, and these relationships have the potential to positively or negatively impact on a person's sense of dignity. Negative consequences can result from being disregarded, for example^44^, ^53^ a situation that many people with dementia can experience. In this study, the facilitators, where possible, interacted with the participants to talk about the questions, to provide explanations and to give positive feedback to them. These were seen as examples of enhancing the engagement of the participants. Hellström et al^43^ argue that establishing good relationships is particularly significant in studies involving people with dementia. Time is needed to build a relationship based on trust and empathy, which may reduce power inequalities.^54^ This corresponds to Morbey et al,^42^ who found it important to have a flexible, responsive and adaptive approach, when involving people with dementia in research. Even sharing a cup of coffee was a way to establish this relationship, as facilitators in this study experienced.

The findings from this paper suggest ways that people with dementia can be supported through the assessment. In Morbey et al’s^42^ study, it became clear that visuospatial abilities, word finding or object recognition difficulties influenced what information was accessible for people with dementia. The researchers had to adjust how they presented the accessible statements by, for example, reading some of these to clarify meaning. These adaptive approaches were echoed in this study as facilitation was matched to individual needs and responses. This flexibility in facilitation is an important way for people with dementia to be part of such research projects and to feel supported in responding as accurately as possible. This highlights that awareness of how dementia symptoms may affect involvement, being relaxed and being flexible in the moment is important aspects of conducting research with people with dementia.^55^ Training may therefore be needed as researchers may be faced with distress, anxiety, grief and declining health when involving people with dementia in research.^56^, ^57^, ^58^ This ensures researchers perform effectively in research.^42^, ^59^

## 6 LIMITATIONS

A limitation is that only ten videos were analysed in this pilot analysis. Thus, it is not possible to generalize these findings. However, the study highlights areas for future research, such as role of facilitation and its potential impact on outcomes. Another limitation is the difference in how facilitation was provided due to the facilitators’ experience and knowledge in dementia research. More training in the measures and how to facilitate the process might have improved the quality of the study.

## 7 CONCLUSION

This study identifies the importance of careful facilitation when involving people with dementia, illustrating, the challenges and dilemmas that might occur during an assessment and how person‐centred facilitation supports participation. This paper concludes that the quality of facilitation may influence the participant, the assessment and the answers given. It is therefore important that the facilitation role is thoroughly planned and executed with ethical consideration as its foundation.

## CONFLICT OF INTERESTS

The authors declare that there is no conflict of interest.

## Data Availability

Anonymized data are available on request due to privacy/ethical restrictions.

## References

[1] Emilson UM. The Staff´s view on dementia and the care in three cultures: a qualitative study in France, Portugal and Sweden. Dementia. 2011;11(1):31‐47. 10.1177/14713012111416613

[2] Berg‐Weger M, Stewart DB. Non‐pharmacologic interventions for persons with dementia. Mo Med. 2017;114(2):116‐119. 30228557 PMC6140014

[3] Cammisuli DM, Danit S, Bosinelli F, Cipriani G. Non‐pharmacological interventions for people with Alzheimer’s disease: a critical review of the scientific literature from the last ten years. Eur Geriatr Med. 2016;7:57‐64.

[4] Harding AJE, Morbey H, Ahmed F, et al. What is important to people living with dementia?: The ‘long‐list’ of outcome items in the development of a core outcome set for use in the evaluation of non‐pharmacological community‐based health and social care interventions. BMC Geriatr. 2019;19(1):94. 10.1186/s12877-019-1103-5 30917790 PMC6437989

[5] Webster L, Groskreutz D, Grinbergs‐Saull A, et al. Core outcome measures for interventions to prevent or slow the progress of dementia for people living with mild to moderate dementia: Systematic review and consensus recommendations. PLoS One. 2017;12(6):e0179521. 10.1371/journal.pone.0179521 28662127 PMC5491018

[6] Orrell M, Spector A, Thorgrimsen L, Woods RT. A pilot study examining the effectiveness of maintenance cognitive stimulation therapy (MCST) for people with dementia. Int J Geriatr Psychiatry. 2005;20(5):446‐451. 10.1002/gps.1304 15852436

[7] Tárraga L, Boada M, Modinos G, et al. A randomised pilot study to assess the efficacy of an interactive, multimedia tool of cognitive stimulation in Alzheimer's disease. J Neurol Neurosurg Psychiatry. 2006;77(10):1116‐1121. 10.1136/jnnp.2005.086074 16820420 PMC2077529

[8] Viola LF, Nunes PV, Yassuda MS, et al. Effects of a multidisciplinary cognitive rehabilitation program for patients with mild Alzheimer's disease. Clinics. 2011;66(8):1395‐1400. 10.1590/S1807-59322011000800015 21915490 PMC3161218

[9] Middlestadt J, Folkerts A‐K, Blawath S, Kalbe E. Cognitive stimulation for people with dementia in long‐term care facilities: baseline cognitive level predicts cognitive gains, moderated by depression. J Alzheimers Dis. 2016;4(1):253‐268. 10.3233/JAD-160181 27497474

[10] Cove J, Jacobi N, Donovan H, Orrell M, Stott J, Spector A. Effectiveness of weekly cognitive stimulation therapy for people with dementia and the additional impact of enhancing cognitive stimulation therapy with a carer training program. Clin Interv Aging. 2014;4(9):2143‐2150. 10.2147/CIA.S66232 PMC4267515 25525349

[11] Mapelli D, di Rosa E , Nocita R, Sava D. Cognitive stimulation in patients with dementia: randomized controlled trial. Dement Geriatr Cogn Disord Extra. 2013;3(1):263‐271. 10.1159/000353457 PMC3776449 24052800

[12] Spector A, Gardner C, Orrell M. The impact of Cognitive Stimulation Therapy groups on people with dementia: views from participants, their carers and group facilitators. Aging Ment Health. 2011;15(8):945‐949. 10.1080/13607863.2011.586622 21722044

[13] Liu Q, Jones M, Hocking C. Describing and measuring the ‘switch‐on’ effect in people with dementia who participate in cognitive stimulation therapy: a mixed methods study. Br J Occup Ther. 2020;83(5):316–325. 10.1177/0308022619899301

[14] Harrison JK, Noel‐Storr AH, Demeyere N, Reynish EL, Quinn TJ. Outcomes measures in a decade of dementia and mild cognitive impairment trials. Alzheimers Res Therapy. 2016;8:48. 10.1186/s13195-016-0216-8 PMC5116815 27866472

[15] Alzheimer’s Society. 2021. https://www.alzheimers.org.uk/about‐dementia/symptoms‐and‐diagnosis/how‐dementia‐progresses/mental‐and‐physical‐activities. Accessed June 6, 2021.

[16] Spector A, Orrell M, Woods RT. Cognitive stimulation therapy (CST): effects on different areas of cognitive function for people with dementia. Int J Geriatr Psychiatry. 2010;25(12):1253‐1258. 10.1002/gps.2464 20069533

[17] Orrell M, Aguirre E, Spector A, et al. Maintenance cognitive stimulation therapy for dementia: single‐blind, multicentre, pragmatic randomised controlled trial. Br J Psychiatry. 2014;204(6):454‐461. 10.1192/bjp.bp.113.137414 24676963

[18] Thoft DS, Ward A, Youell J. Journey of ethics – conducting collaborative research with people with dementia. Dementia. 2021;20(3):1005‐1024. 10.1177/1471301220919887 32326751

[19] Ward A, Thoft DS, Soerensen AL. A narrative literature review of quantitative and qualitative approaches used to explore the use of outcome measures with cognitive stimulation therapy, cognitive training, and cognitive stimulation interventions, and how these relate to the experiences of people with dementia. Dementia (in review 2020).

[20] Hall L, Orrell M, Stott J, Spector A. Cognitive stimulation therapy (CST): neuropsychological mechanisms of change. Int Psychogeriatr. 2013;25(3):479‐489. 10.1017/S1041610212001822 23146408

[21] Bergamaschi S, Arcara G, Calza A, Villani D, Orgeta V, Mondini S. One‐year repeated cycles of cognitive training (CT) for Alzheimer's disease. Aging Clin Exp Res. 2013;25(4):421‐426. 10.1007/s40520-013-0065-2 23784727

[22] Thoft DS, Pyer M, Horsbøl A, Parkes J. The Balanced Participation Model: sharing opportunities for giving people with early‐stage dementia a voice in research. Dementia. 2020;19(7):2294–2313. 10.1177/1471301218820208 30587030

[23] Thoft DS. Involving people with early‐stage dementia in qualitative research about their lifeworld perspectives: Development of a participatory research model. University of Northampton; 2017.

[24] Ward A, Schack Thoft D, Lomax H, Parkes J. A visual and creative approach to exploring people with dementia’s experiences of being students at a school in Denmark. Dementia. 2020;19(3):786–804. 10.1177/1471301218786636 29999411

[25] Ward A. Understanding photography and storytelling with people with early‐stage dementia to understand their lived experience and enable them to tell their stories. University of Northampton; 2019.

[26] Ward A, Alberg Sorensen K, Kousgaard H, Schack Thoft D, Parkes J. Going back to school – an opportunity for lifelong learning for people with dementia in Denmark (Innovative practice). Dementia. 2020;19(7):2461–2468. 10.1177/1471301218763190 29528700

[27] Folstein MF, Folstein SE, McHugh PR. ‘‘Mini‐mental state’’: a practical method for grading the cognitive state of patients for the clinician. J Psychiatr Res. 1975;12:189‐198. 10.1016/0022-3956(75)90026-6 1202204

[28] Psychological Assessment Resources. MMSE‐2nd Edition Blue print; 2010. https://www.parinc.com/Products/Pkey/238. Accessed June 6, 2021.

[29] Logsdon R, Gibbons LE, McCurry SM, Teri L. Quality of life in Alzheimer’s disease: patient and caregiver reports. J Mental Health Aging. 1999;5:21‐32.

[30] Logsdon RG, Gibbons LE, McCurry SM, Teri L. Assessing quality of life in older adults with cognitive impairment. Psychosom Med. 2002;64:510‐519. 10.1097/00006842-200205000-00016 12021425

[31] Jerusalem M, Schwarzer R, General Self‐Efficacy Scale (GSE) . 1981. https://userpage.fu‐berlin.de/~health/selfscal.htm. Accessed June 6, 2021.

[32] Rosenberg M. Society and the Adolescent Self‐Image. Princeton, NJ: Princeton University Press; 1965.

[33] Hawthorne G. Measuring social isolation in older adults: development and initial validation of the Friendship Scale. Soc Indic Res. 2006;77:521‐548. 10.1007/s11205-005-7746-y

[34] Ridder AM. Microanalysis on selected video clips with focus on communicative response in music therapy. In: Wosch T, Wigram T, eds. Microanalysis in Music Therapy: Methods, Techniques and Applications for Clinicians. Jessica Kingsley Publishers; 2007:54–66.

[35] Lomax H, Casey N. Recording Social Life: Reflexivity and Video Methodology. Sociological Res Online. 1998;3(2):121–146. 10.5153/sro.1372

[36] Derry SJ, Pea RD, Barron B, et al. Conducting video research in the learning sciences: guidance on selection, analysis, technology, and ethics. J Learn Sci. 2010;19(1):3‐53. 10.1080/10508400903452884

[37] Erickson F. Definition and analysis of data from videotape: Some research procedures and their rationales. In: Green JL, Camilli G, Elmore PB, eds. Handbook of Complementary Methods in Education Research. Mahwah, NJ: Erlbaum; 2006:177‐205.

[38] World Medical Association. Declaration of Helsinki . 1964. https://www.wma.net/wp‐content/uploads/2018/07/DoH‐Jun1964.pdf. Accessed June 6, 2021.

[39] Dewing J. Participatory research: a method for process consent with persons who have dementia. Dementia. 2007;6(1):11‐25. 10.1177/1471301207075625

[40] Folstein MF, Folstein SE, Mchugh PR, Fanjiang G. MMSE Mini‐Mental State Examination. Users Guide. Lutz, F1.: PAR. Psychological Assessment Resources, Inc.; 2001. https://www.parinc.com/Products/Pkey/237. Accessed June 6, 2021.

[41] Demensrådet . Mini‐Mental Status Examination (MMSE). Vejledning i administration og scoring for RegionH 2010.

[42] Morbey H, Harding AJE, Swarbrick C, et al. Involving people living with dementia in research: an accessible modified Delphi survey for core outcome set development. Trials. 2019;12(1):1–10. 10.1186/s13063-018-3069-6 PMC6322337 30612587

[43] Hellström I, Nolan M, Nordenfelt L, Lundh U. Ethical and methodological issues in interviewing persons with dementia. Nurs Ethics. 2007;14(5):608‐619. 10.1177/0969733007080206 17901172

[44] Heggestad AKT, Nortvedt P, Slettebø Å. The importance of moral sensitivity when including persons with dementia in qualitative research. Nurs Ethics. 2012;20(1):30‐40. 10.1177/0969733012455564 23166145

[45] Nordenfelt L. The varieties of dignity. Health Care Anal. 2004;12(2):69‐82; discussion 83‐89. 10.1023/B:HCAN.0000041183.78435.4b 15487812

[46] Fisk M, Wigley V. Accessing and interviewing the oldest old in care homes. Quality Age Older Adults. 2000;1(1):27‐33. 10.1108/14717794200000005

[47] Howe E. Informed consent, participation in research, and the Alzheimer´s patient. Innov Clin Neurosci. 2012;9(5‐6):47‐51. PMC3398682 22808449

[48] Lacy M, Kaemmerer T, Czipri S. Standardized mini‐mental state examination scores and verbal memory performance at a memory center. Am J Alzheimer' Dis Other Demen. 2015;30(2):145–152. 10.1177/1533317514539378 PMC10852598 24990889

[49] Fisher P. Ethics in qualitative research: ‘Vulnerability’, Citizenship and Human Rights. Ethic Soc Welfare. 2012;6(1):2‐17. 10.1080/17496535.2011.591811

[50] Cotrell V, Schulz R. The perspective of the patient with Alzheimer’s disease: a neglected dimension of dementia research. Gerontologist. 1993;33(2):205‐211. 10.1093/geront/33.2.205 8468013

[51] Edlund M. Människans värdighet: ett grundbegrepp inom vårdvetenskapen (Human Dignity – A Basic Caring Science Concept). Åbo: Åbo Akademis förlag; 2002.

[52] Nordenfelt L. The concept of dignity. In: Nordenfelt L ed. Dignity in Care for Older People. 1st edn. Chichester: Wiley‐Blackwell; 2009:26‐53.

[53] Jacobson N. A taxonomy of dignity: a grounded theory study. BMC Int Health Hum Rights. 2009;9:1‐9. 10.1186/1472-698X-9-3 19239684 PMC2656457

[54] Wilkinson H. Including people with dementia in research: methods and motivations. In: Wilkinson H, ed. The Perspectives of People with Dementia: Research Methods and Motivations. London: Jessica Kingsley; 2002:9‐24. 10.7748/nop.14.3.36.s22 27316366

[55] Thoft DS, Ward A, Youell J. Journey of ethics – conducting collaborative research with people with dementia. Dementia. 2021;20(3):1005–1024. 10.1177/1471301220919887 32326751

[56] Dickson‐Swift V, James EL, Kippen S, Liamputtong P. Researching sensitive topics: qualitative research as emotion work. Qual Res. 2009;9(1):61‐79. 10.1177/1468794108098031

[57] Kumar S, Cavallaro L. Researcher self‐care in emotionally demanding research: a proposed conceptual framework. Qual Health Res. 2018;28(4):648‐658. 10.1177/1049732317746377 29224510

[58] McGarrol S. The emotional challenges of conducting in‐depth research into significant health issues in health geography: reflections on emotional labour, fieldwork and life course. Area (Oxf). 2017;49(4):436‐442. 10.1111/area.12347 29400349 PMC5765835

[59] Elvish R, Cawley R, Keady J. The experiences of therapy from the perspectives of carers of people with dementia: an exploratory study. Couns Psychother Res. 2013;14(1):56‐63. 10.1080/14733145.2013.768284

